# Kawasaki disease involving both the nervous system and cardiovascular system: a case report and literature review

**DOI:** 10.3389/fped.2024.1459143

**Published:** 2024-12-02

**Authors:** Wen Yin, Yali Wu, Shasha Wang, Hongxia Tang, Yan Ding

**Affiliations:** Department of Rheumatology and Immunology, Wuhan Children’s Hospital (Wuhan Maternal and Child Healthcare Hospital), Tongji Medical College, Huazhong University of Science & Technology, Wuhan, China

**Keywords:** Kawasaki disease, coronary artery lesions, facial nerve paralysis, ptosis, systemic artery aneurysms

## Abstract

**Background:**

Kawasaki disease (KD), an acute self-limiting vasculitis, is the main cause of acquired heart disease. Timely diagnosis and treatment can mitigate the occurrence of complications and improve patient prognosis. Facial nerve paralysis (FNP) and ptosis are rare complications of KD and are rarely reported, but FNP is considered a high risk factor for coronary aneurysms. If giant coronary artery aneurysms are formed, clinicians should be vigilant when diagnosing the formation of systemic artery aneurysms (SAAs).

**Patient presentation:**

A 10-month-old girl with fever, diffuse rash, and conjunctival congestion was hospitalized locally, diagnosed with KD, and treated with intravenous infusion of gamma globulin (IVIG). She had fever again after 1 week of temperature stability, accompanied by conjunctival congestion, and was treated with a second dose of IVIG, but she still experienced recurrent fever. The day prior to admission, she developed facial asymmetry, left FNP, diffuse erythema and membranous peeling of the fingers of both hands. The patient's body temperature normalized after treatment with 20 mg/kg methylprednisolone, but cardiac ultrasound revealed progressive enlargement of the coronary artery aneurysms. On day 37of the illness, transient eyelid ptosis developed; fortunately, the left FNP and eyelid ptosis finally resolved, leaving no sequelae. Two years and eight months after onset, the patient developed bilateral humeral aneurysm.

**Conclusion:**

This is the first KD patient involving two neurological complications combined with giant coronary artery aneurysms and SAAs. KD needs to be considered in infants with unexplained recurrent fever who present with FNP or ptosis. FNP secondary to KD is a high risk factor for coronary artery aneurysms, so it is necessary to perform cardiac ultrasound for accurate diagnosis. KDs combined with giant coronary aneurysms require careful physical examination and noninvasive angiography during follow-up to detect SAA formation.

## Introduction

Kawasaki disease (KD) is an acute self-limiting vasculitis disease that primarily affects children under 5 years of age, and the pathogenesis of KD has remained unclear until now. Coronary artery lesions (CALs) are serious complications of KD that can cause sudden death, myocardial infarction and ischemic heart disease in children and are now the leading cause of acquired heart disease in children. Timely diagnosis and treatment with intravenous infusion of gamma globulin (IVIG) reduce the probability of CALs from 25% to approximately 4%. The extent of coronary artery involvement is a crucial factor affecting the long-term prognosis of KD patients (1, 2). It is well known that KD causes systemic inflammation in all medium-sized arteries, and the incidence of systemic artery aneurysms (SAAs), including those of the subclavian, brachial, axillary, and iliac arteries, is approximately 0.8%–2.0% (3). In addition to affecting the cardiovascular system, KD can also cause complications in other organs and tissues. The incidence of neurological complications is approximately 1%–30% (4, 5), including headache, epilepsy, lethargy, irritability, aseptic meningitis, bulging fontanel, facial nerve paralysis (FNP) and sensorineural hearing loss (6). The incidence of KD combined with FNP is only 0.9%–1.3% (7). KD combined with bilateral ptosis is even rarer, with only 6 cases reported thus far (8–13). We report a patient with KD with FNP, transient bilateral ptosis and giant coronary artery aneurysms. Bilateral brachial artery aneurysms were found after 2 years and 8 months of follow-up.

## Case

This was a retrospective study approved by the Institutional Review Board of Wuhan Children's Hospital, Tongji Medical College, Huazhong University of Science and Technology (No. 2021R074-E02).

A 10-month-old female infant who was diagnosed with KD at a local hospital after infection was ruled out and given IVIG at a dose of 2 g/kg and aspirin (30 mg/kg/day) treatment due to fever for 1 week accompanied by generalized erythema, conjunctival hyperemia and chapped lips. After stabilizing her temperature for one week, her fever recurred with bilateral conjunctival congestion, without rash, and without symptoms related to infection such as cough or rhinorrhea, and she subsequently received a second dose of 2 g/kg IVIG at the same local hospital, but the fever still recurred. On the 20th day of recurrent fever, facial asymmetry appeared, the left nasolabial sulcus was shallowed, and the left eye could not be completely closed; therefore, he was admitted to our hospital.

Physical examination on admission, which revealed bilateral bulbar conjunctival congestion, strawberry tongue, fissuring of the lips, swollen lymph nodes in the neck, erythematous rash scattered throughout the body, incomplete closure of the left eyelid, corners of the mouth inclined to the right, shallow left nasolabial furrows, and membranous peeling on the fingers of both hands. Thus, IVIG-resistant KD was diagnosed.

**Figure 1:**
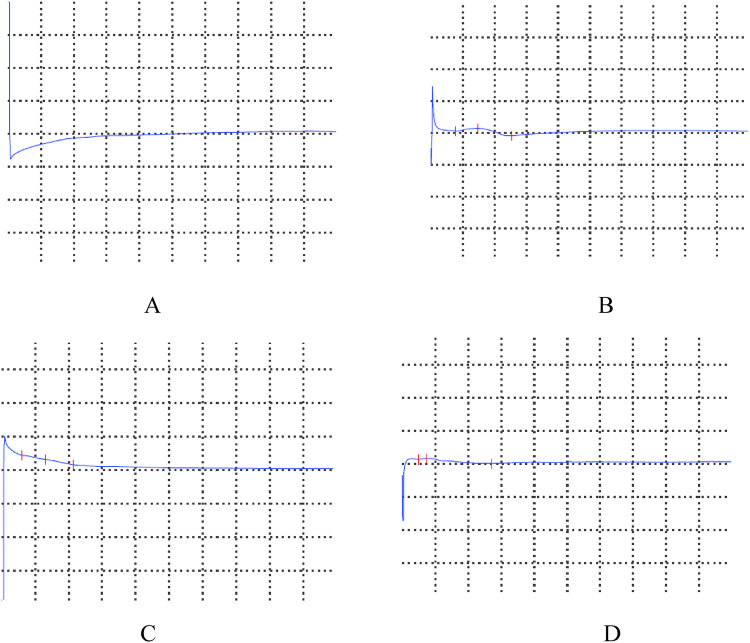
Electromyogram: facial nerve conduction velocity. Normal conduction velocity of the right facial nerve. No definitive compound muscle action potential was evoked in the left facial nerve motor conduction study with recording from the orbicularis oris muscle. **(A)** Left facial nerve - orbicularis oris muscle. **(B)** Right facial nerve - orbicularis oris muscle. **(C)** Left facial nerve - orbicularis oculi. **(D)** Right facial nerve - orbicularis oculi.

**Figure 2:**
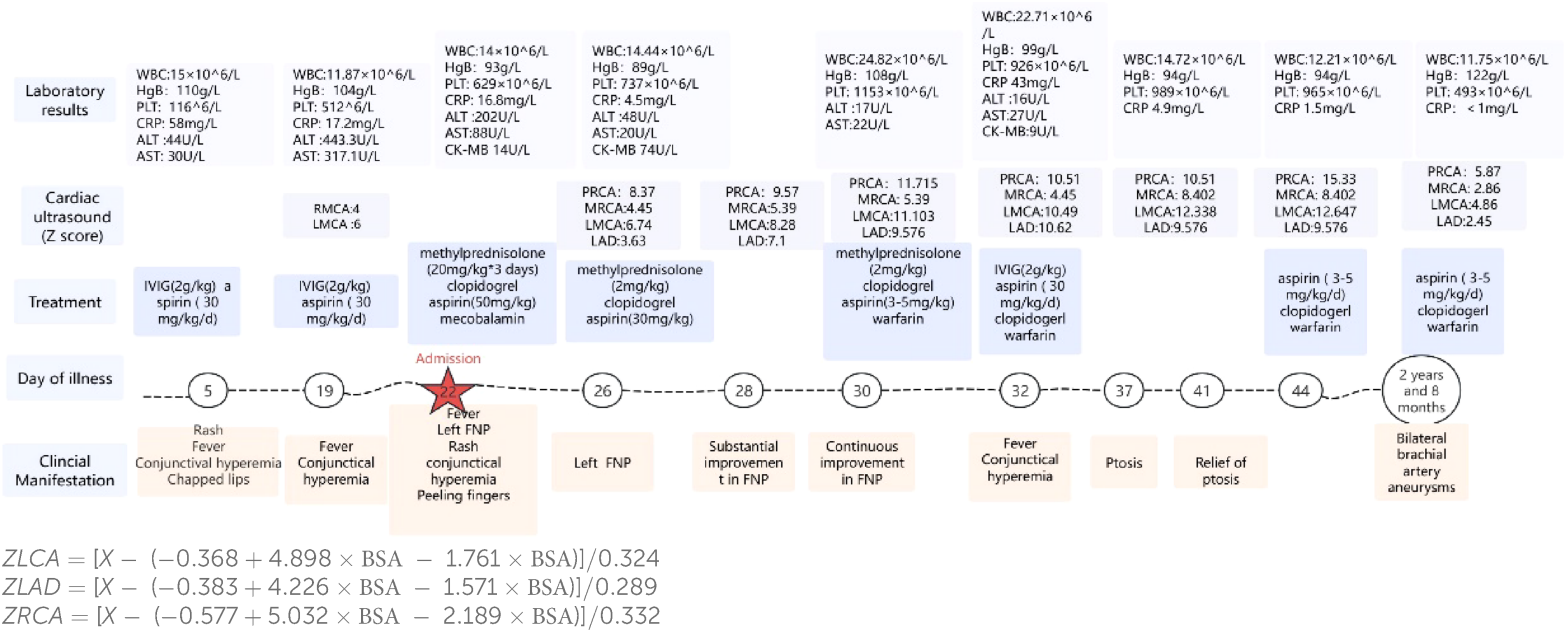
A progress chart presenting historical findings and treatment over time. PRCA: proximal segment of the right coronary artery. MRCA: middle segment of the right coronary artery. RMCA, left main coronary artery; LMCA, left main coronary artery; LAD, left anterior descending; WBC, white blood cell; HgB, hemoglobin; PLT, platelet; CRP, C-reactive protein; ALT, alanine aminotransferase; AST, aspartate aminotransferase; CK-MB, creatine kinase-MB.

Cephalic magnetic resonance and electroencephalogram results were normal. Electromyography suggested a lesion in the left peripheral facial nerve (Figure 1). Her white blood cell count was 14.00 × 10^9^/L, hemoglobin level was 93 g/L, platelet count was 629 × 10^9^/L, C-reactive protein level was 16.80 mg/L, and her interleukin-6 level was 164.95 pg/mL. Testing for antibodies to EB virus, cytomegalovirus, herpes simplex virus type I, and herpes simplex virus type II has ruled out infection. Routine analysis of cerebrospinal fluid revealed a white blood cell count of 21 × 10^6^/L (reference range 0–15 × 10^6^/L), dominant mononuclear cells, a cerebrospinal fluid protein level of 0.50 (reference range 0.05–0.45 g/L), and a cerebrospinal fluid glucose level of 4.09 (2.2–3.9 mmol/L). After treatment with methylprednisolone (20 mg/kg) for 3 days, aspirin (50 mg/kg/day) and mecobalamin were administered. After the infusion of methylprednisolone, her temperature normalized. The remaining FNP was alleviated after 1 week. After discharge, prednisone, clopidogrel, warfarin, and aspirin were continued. Her fever reappeared on the 32th day with conjunctival congestion, and she was admitted to our department for treatment again. Her temperature returned to normal after IVIG (2 kg/kg) treatment, but on the 37th day after disease progression, this child presented with bilateral ptosis, but muscle strength level 5, and the knee-jerk reflex can be normally elicited. Unlike myasthenia gravis, this child's bilateral ptosis was obvious in the morning, relatively relieved in the afternoon, and persisted for 5 days, and this phenomenon was largely relieved. Ganglioside antibody testing revealed positivity for anti-GD2 IgM and anti-GM2 IgM. After a follow-up period of 2 years and 8 months, the parents discovered masses in both elbows, while bilateral brachial aneurysms were indicated by cardiac CT angiography. A progress chart presenting historical findings and treatment over time in Figure 2 (14).

## Discussion

KD is a unique syndrome characterized by persistent fever, conjunctival congestion, fissured lips or strawberries, erythema or edema of the hands and feet, cervical lymphadenopathy and polymorphous rash (15, 16). CALs are serious complications of KD, and the risk of coronary artery aneurysm in infants less than 1 year old is significantly increased (17). In addition, IVIG resistance is also a risk factor for coronary aneurysms. A single-center retrospective study in Italy revealed that approximately 50% of children under 1 year of age with KD had coronary artery lesions and increased IVIG resistance (18).

Although the incidence of neurological complications associated with KD is not high, neurological complications such as irritability, aseptic meningitis, facial nerve paralysis, and sensorineural hearing loss have been recognized as neurological manifestations of KD (1). To date, there have been a few cases of KD combined with FNP. This is the first patient with unilateral FNP and bilateral ptosis accompanied by bilateral giant coronary aneurysms and bilateral brachial aneurysms during follow-up. We summarized the clinical features and prognosis of 19 patients with KD complicated with FNPs since 2018 (Table 1). Seventy-nine percent of the patients were under 1 year old, twelve patients had left FNP, the median onset time of facial nerve palsy was the 11th day of illness, 18 out of 19 patients had coronary artery lesions, and the proportion of coronary artery aneurysms was 68%. According to the article published by Stowe, FNP secondary to KD mainly affects infants under 1 year of age (63.9%), and the onset time is 16 days in the course of the disease. Left FNP is more common, and coronary artery aneurysms are observed in 24 out of 36 KD patients with FNP (28). Our two research conclusions are basically consistent. Therefore, FNP in KD patients may be a high risk factor for severe coronary artery aneurysm (20). Histopathological examination of the nervous system in children with KD revealed ganglionitis and neuritis of cranial and peripheral nerves, so it is believed that ischemic vasculitis supplying the facial nerve is the cause of FNP (4). In addition, we found that in 6 patients, including our infants, FNP occurred following the administration of IVIG, and all of these FNPs were associated with CALs. Therefore, we believe that an excessive inflammatory response is also involved in the occurrence of FNP (21, 27). However, the prognosis of FNP secondary to KD is not bad, and it can be completely alleviated except for 2 patients with bilateral nerve paralysis (23, 29).

**Table 1:** The clinical features and prognosis of 19 patients with KD complicated with FNPs since 2018.

Author	Age (month)	Sex	Affected side	Day of FNP onset	CA	FNP onset related to IVIG use	Outcome of FNP and CA
Ours	10	F	L	20	Bilateral giant CAA	After	FNP relieved after 1 week. CAA return to small CA with a maximum *Z* score of 5.87 in 2.8year
Murata et al. (19)	4	F	L	6	Right giant CAA, left small-size CAA	Before	FNP complete improved 6 weeks, CAA were improved somewhat but remained two years after onset
Maglione et al. (20)	4	M	R	6	Bilateral small to medium-size CAA	Before	FNP relieved after 23 days, improvement of right CA with a mild dilatation (2.8 mm, *z* score 5), complete resolution of left CA after 3 month
Peña-Juárez et al. (21)	9	F	L	29	Bilateral giant CAA	After	FNP resolved after 1 week persistence of the giant CAA after 1year
Yuan and Lu (22)	4	F	R	7	Right giant CAA, left small to medium-size CAA	Before	FNP resolved after 20 days after onset. improvement of CA after 3months
Zhang et al. (23)	6	M	L+R	6	Bilateral CAA	Before	The right FNP was relieved 1 month, left FNP and CAA persisted after 18-month
Yuan and Lu (24)	3	M	L	8	Left CA dilatation	Before	Full recovery of FNP at 14 days after onset, left CA returned to normal
Rodriguez-Gonzalez et al. (25)	5	M	L	10	Bilateral small to medium-size CAA	Before	FNP resolved completely after 3 days, CAA subsided slightly after 6 months
Orgun et al. (26)	4	F	L	7	Bilateral medium-size CAA	Before	FNP recovered after 7 days CAA has continued with minimal improvement after 3 months
Chen et al. (27)	8	M	L	6	Normal	Before	FNP recovered after 27 days
Chen et al. (27)	3	F	L	6	Bilateral dilation	Before	FNP recovered after 57 days
Chen et al. (27)	8	M	L	10	Bilateral dilation	After	FNP recovered after 10 days
Chen et al. (27)	13	M	L	16	Right medium-size CAA	Before	FNP recovered after15 days
Chen et al. (27)	4	F	R	10	Bilateral dilation	Before	FNP recovered after 11 days
Chen et al. (27)	24	F	L	11	Bilateral medium-size CAA	After	FNP recovered after 25 days
Chen et al. (27)	5	M	L	8	Bilateral small-size CAA	Before	FNP recovered after 36 days
Chen et al. (27)	108	M	L+R	15	Bilateral dilation	After	FNP recovered after 19 days
Chen et al. (27)	5	F	R	11	Left small-size CAA	After	FNP recovered after 130 days

Ptosis secondary to KD was rarer, with only 6 cases in the early stage, none of which were complicated with CALs. Regardless of whether it occurs in the acute or subacute phase of the disease course, the symptoms of ptosis can be completely relieved (9–12). The mechanism of ptosis in KD remains unclear, and Falcini et al. suggested that it may be caused by ischemic vasculitis supplying the elevator muscles of the palpebra (9). Sanchez Marcos's study revealed positivity for anti-acetylcholine receptor antibodies (12), but laboratory tests in our child revealed the presence of ganglioside antibodies. IgM anti-GM2 or GD2 antibodies are rare types of antiganglioside antibodies that can be detected in Guillain-Barré syndrome (GBS). GBS is also an immune-mediated acute polyradiculoneuropathy. Studies have indicated that IgM anti-GM2 antibodies are associated with facial weakness in patients with GBS (30), in our case, the patient presented with bilateral ptosis. Therefore, we hypothesized that this KD child also had a variant of GBS. Regrettably, we did not repeat the examination of cerebrospinal fluid, electromyography, and cranial magnetic resonance imaging to confirm.

**Figure 3:**
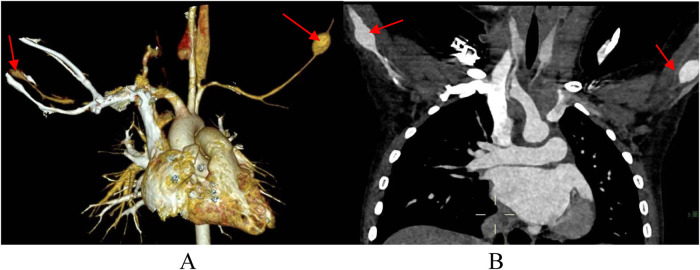
Cardiac CT angiography at 2 years and 8 months of follow-up. **(A,B)** arrows indicate bilateral brachial aneurysms.

There are few studies on the risk factors, incidence and prognosis of SAAs in KD patients. Zhao et al. suggested that the incidence of KD combined with SAA is approximately 2%, and this group of patients all have medium-sized coronary aneurysms with a *Z* value >8, 73.9% (17 of 23) of which are giant CAAs; in addition, the occurrence of SAAs is generally symmetrical (31). Similar to the prognosis of coronary aneurysms, the regression rate of SAAs is related to artery diameter, and an SAA diameter >10 mm in the acute stage indicates that stenosis may occur in the later stage (32). The patient we reported had a palpable mass in both elbows during physical examination and bilateral brachial aneurysms with a diameter of 12 mm by cardiac CT angiography at 2 years and 8 months of follow-up after disease onset (Figure 3). Therefore, noninvasive imaging modalities should be used to screen for SAAs within 2 months of onset in children with giant coronary aneurysms. During the recovery phase, patients are followed up by peripheral angiography. The duration of follow-up was determined by the extent of coronary artery damage (31).

## Conclusion

Both FNP and bilateral ptosis are rare complications of KD, so the presence of FNP or transient ptosis in children with unexplained prolonged fever should be considered for KD, especially in infants under 1 year of age. Different from previous cases, our patient developed unilateral FNP and transient ptosis in a short period of time, with progressive enlargement of the coronary aneurysm. After these two symptoms are basically relieved, the coronary aneurysm also began to gradually recover. Therefore, we believe that the more neurological complications associated with Kawasaki disease, the more intense the inflammatory response, and long-term follow-up is necessary to be vigilant for the formation of SAA. Fortunately, these two neurological complications have good prognoses and few sequelae.

## Data Availability

The original contributions presented in the study are included in the article/Supplementary Material, further inquiries can be directed to the corresponding authors.
